# Editorial: Moving the needle on children's physical activity – How to best promote more movement?

**DOI:** 10.3389/fpubh.2024.1454223

**Published:** 2024-07-12

**Authors:** Jennifer M. Sacheck, Mirko Brandes

**Affiliations:** ^1^Department of Exercise and Nutrition Sciences, Milken Institute School of Public Health, The George Washington University, Washington, DC, United States; ^2^Department of Prevention and Evaluation, Leibniz Institute for Prevention Research and Epidemiology (LG), Bremen, Germany

**Keywords:** physical activity, physical literacy, school-aged children, sedentary time, moderate to vigorous physical activity

In this Research Topic, we highlight research and innovative ways in which we can work toward moving the needle on children's physical activity. Globally, the vast majority of children do not meet the World Health Organization's recommendations for daily moderate to vigorous physical activity (MVPA) while at the same time children have become increasingly sedentary (1).

To increase children's engagement in physical activity, this Research Topic includes studies and discussions on school-based interventions (and engaging the home), the broader “context” of the intervention needing to be considered for interventions to be effective, and the need for additional focus on personal attributes such as fundamental movement skills, self-efficacy, and fitness to ensure children can and will engage in physical activity. Not surprisingly, articles in this Research Topic have focused on MVPA and sedentary time, largely in the school setting where children, on average, spend the a great deal of their time and where many interventions have focused on increasing children's PA. Al-walah et al. presented pilot data on a randomized controlled trial (RCT) implemented in Saudi-Arabian pre-schools that targeted both MVPA and sedentary time and attempted to engage the home environment. Implementation was largely successful (with noted challenges in engaging the home), however they were not able to increase PA or decrease sedentary time.

These results are not surprising to many PA researchers. RCTs are still largely considered the gold standard for research funding in this space, even though implementation of such interventions in the school setting is extremely challenging. This point is further demonstrated by the scoping review by Porter et al. and the accompanying commentary by Jago et al. where the authors discuss the limitations of examining PA interventions in the RCT format. They highlight that the community-, school-, and population-specific “contexts” are not often considered, instead rigid research protocols are favored to maintain internal validity (which makes funding agencies and peer-reviewers feel more confident in the possibility of success of an intervention). To overcome this limitation for successful promotion of physical activity in the real world, the authors developed a framework for the design of more tailored interventions but did put forth that interventions in their review (whether successful or not) often did not include context-specific details that would help promote success in this area.

Other articles in this Research Topic also supported the need for consideration of context of the intervention environment. St. Pierre et al. discussed the effectiveness of utilizing “near-peer” coaches in the middle school setting in low-income schools of New Orleans, LA. They discuss how the relatability of these coaches with the kids in the PA intervention trial made it more meaningful amongst a population of youth where consideration of PA and related health outcomes may need to be reconstructed due to competing priorities (e.g., staying in school, poverty). Broader context outside of the school day was also considered by Pfledderer et al., where they reinforced the importance of out-of-school physical activity (organized sports and activities, outdoor play, etc.) on children meeting PA guidelines in a large sample of youth who completed the Texas-SPAN survey.

Beyond the intervention environment, within child context should be considered as well. A re-emerging focus on children's physical literacy (PL), “the competence, confidence, and motivation to be physically active” (2, 3), is another key aspect to foster to ensure that children are physically able to and mentally want to engage in physical activities. Importantly, PL has been shown to be associated with greater levels of PA and as demonstrated by Chai et al. Furthermore, Grauduszus et al. conducted a scoping review of school-based PL interventions which emphasized the growing literature base in this space. There remains, however, a lack of consistent methodologies for measurement of PL as well as variable PA outcomes as a result of PL interventions, again speaking to the need for tailoring interventions to context as well as utilizing methodologies that will enable researchers to discern PL outcomes. PL and PA interventions should also consider the personal characteristics of the individual/child participant (beyond demographics) such as physical fitness levels. Graham et al. demonstrated how children and youth with varying levels of fitness may differentially respond to PA interventions targeting improving PA and related health outcomes.

Additionally, when looking broadly at interventions that have been or can be the most successful among children, two articles in this Research Topic have highlighted the importance of intervening across multiple levels of the social ecological model to ensure greater likelihood of increasing PA among children and youth (Sell et al.; Cholley-Gomez et al.). This ideal is not without its logistical and practical challenges.

Indeed, many school-based interventions are now also targeting the home environment (or family) given the importance of these two environments being key in supporting PA of children. Even within these two areas of influence, there are many aspects to consider in what could help change a child's physical activity patterns. It is also challenging to best measure intervention implementation so that changes in PA are detectable. Finally, all movement should be considered. We should not disregard or not attempt to measure light physical activity in children, even though we currently do not (yet) have guidelines for it. We should think of PA as a continuum, and not either “on (MVPA)” or “off (sedentary time)”. Besides issues of how to measure physical activity, we also need to put more emphasis on how to get every child to want to engage in more movement, e.g., by taking up children's motivation (a key component of PL) to be (more) physically active. If we begin to more thoughtfully consider the multitude of ways our children and their environments shape their physical activity patterns and work to make subtle changes in this regard, we can make significant strides in moving the needle on their physical activity.

## Author contributions

JS: Conceptualization, Writing – original draft, Writing – review & editing. MB: Writing – review & editing, Conceptualization.
